# Abdominal Compartment Syndrome and Intra-abdominal Ischemia in Patients with Severe Acute Pancreatitis

**DOI:** 10.1007/s00268-015-3388-7

**Published:** 2016-02-01

**Authors:** M. Smit, K. T. Buddingh, B. Bosma, V. B. Nieuwenhuijs, H. S. Hofker, J. G. Zijlstra

**Affiliations:** Department of Critical Care (BA 49), University Medical Center Groningen, University of Groningen, PO Box 30001, 9700 RB Groningen, The Netherlands; Department of Surgery, University Medical Center Groningen, University of Groningen, Groningen, The Netherlands

## Abstract

**Introduction:**

Severe acute pancreatitis may be complicated by intra-abdominal hypertension (IAH), abdominal compartment syndrome (ACS), and intestinal ischemia. The aim of this retrospective study is to describe the incidence, treatment, and outcome of patients with severe acute pancreatitis and ACS, in particular the occurrence of intestinal ischemia.

**Methods:**

The medical records of all patients admitted with severe acute pancreatitis admitted to the ICU of a tertiary referral center were reviewed. The criteria proposed by the World Society of the Abdominal Compartment Syndrome (WSACS) were used to determine whether patients had IAH or ACS.

**Results:**

Fifty-nine patients with severe acute pancreatitis were identified. Intra-abdominal pressure (IAP) measurements were performed in 29 patients (49.2 %). IAH was present in all patients (29/29). ACS developed in 13/29 (44.8 %) patients. Ten patients with ACS underwent decompressive laparotomy. A large proportion of patients with ACS had intra-abdominal ischemia upon laparotomy: 8/13 (61.5 %). Mortality was high in both the ACS group and the IAH group.

**Conclusion:**

This study confirms that ACS is common in severe acute pancreatitis. Intra-abdominal ischemia occurs in a large proportion of patients with ACS. Swift surgical intervention may be indicated when conservative measures fail in patients with ACS. National and international guidelines need to be updated so that routine IAP measurements become standard of care for patients with severe acute pancreatitis in the ICU.

## Introduction

Acute pancreatitis is a well-established risk factor for intra-abdominal hypertension (IAH) and abdominal compartment syndrome (ACS) [1]. The incidence of IAH in patients with severe acute pancreatitis is approximately 60 % [2, 3], while ACS may occur in approximately 30 % [2]. The mortality of patients with severe acute pancreatitis who develop ACS is high at 50–75 % [2–4].

Respiratory, renal, and cardiovascular organ failures have been reported in patients with ACS. Furthermore, ACS may cause mesenteric hypoperfusion and subsequent ischemia of intra-abdominal organs [5]. Mesenteric ischemia and necrosis may also develop in severe acute pancreatitis [6]. Ischemia–reperfusion injury due in IAH and ACS has mainly been studied in animal models [7–9]. In a retrospective series in 26 children with ACS, 19 % required bowel resection due to ischemic necrosis [10]. Furthermore, raised IAP may be an important mechanism behind colonic hypoperfusion after ruptured aortic aneurysm repair [11]. However, there has not yet been a series detailing the occurrence of mesenteric ischemia in patients with severe acute pancreatitis and ACS.

The aim of this study is to describe the incidence, treatment, and outcome of patients with severe acute pancreatitis and ACS in the intensive care unit (ICU), in particular the occurrence of intestinal ischemia.

## Materials and methods

### Definitions

#### Severe acute pancreatitis (SAP)

Acute pancreatitis with persistent organ failure (>48 h) as defined by the revised Atlanta criteria [12].

#### Multiple organ failure (MOF)

The failure of >1 organ system (respiratory, renal, or cardiovascular) [12].

#### Intra-abdominal hypertension (IAH)

A sustained or repeated pathological elevation in IAP of 12 mmHg or higher [1] categorized into grade I (IAP 12–15 mmHg), grade II (16–20 mmHg), grade III (21–25 mmHg), and grade IV (>25 mmHg).

#### Abdominal compartment Syndrome (ACS)

A sustained IAP >20 mmHg that is associated with new organ dysfunction/failure, as defined by the World Society of the Abdominal Compartment Syndrome (WSACS) [1].

### Patients

This study was conducted at a tertiary referral center for patients with severe acute pancreatitis. Patients were included who met the following criteria: (1) who were admitted to the ICU between January 2005 and May 2011 and (2) who met the revised Atlanta criteria for *severe* acute pancreatitis.

Patients were excluded if they met any of the following criteria: (1) age under 18 years; (2) history of chronic pancreatitis; (3) admission after resuscitation for cardiac arrest; and (4) pancreatitis was diagnosed post-mortem. Furthermore, one patient was excluded because the medical records were incomplete. Medical records of all patients were examined; this included examination of the daily nursing charts where intra-abdominal pressure (IAP) measurements are noted, the admission notes and ICU discharge notes, the discharge notes of the general ward, all surgery reports, and all microbiologic culture reports.

Transvesical IAP measurement was performed according to a standard protocol based on the WSACS consensus definitions [1].

### Statistical analysis

Descriptive statistics were used. Dichotomous data were presented by proportions and continuous data by means (with standard deviations) or medians (with rages) according to normality. Pearson’s *χ*^2^ test, paired and independent Student *T* tests, and Mann–Whitney tests were used as appropriate. Due to small numbers of events, multivariate analysis was deemed inappropriate.

## Results

### Patient characteristics

Fifty-nine patients were identified that met the inclusion criteria. Most patients were males, around 60 years old and referred from a regional hospital. The mean interval between admission to a (regional) hospital and the tertiary ICU was 13.4 days (SD 20.7, range 0–123). The most common etiology was gallstones (40.7 %), followed by alcoholic pancreatitis (22.0 %). Most patients were critically ill; 84.7 % had MOF and 62.7 % had infected pancreatic necrosis at some point during their illness.

### Intra-abdominal pressure

Patient characteristics and IAP measurements are summarized in Table 1. IAP measurement was performed in 29 out of 59 patients (49.2 %). Age and Apache II scores did not differ significantly between the group where IAP was measured, compared to where no IAP measurements were done. The mean interval between admission to a (regional) hospital and the tertiary ICU was 9.3 days in the first group and 17.2 days in the second group.

**Table 1:** Patient characteristics: IAP measurements versus no IAP measurements

	IAP measurements	No IAP measurements	*p* value
Number of patients	29	30	
Female gender	7	16	**0.042**
Age, mean ± SD	59.6 ± 13.9	61.8 ± 13.6	0.557
Apache II score, mean ± SD	26.6 ± 7.3	26.1 ± 11.8	0.849
Interval between hospital admission and tertiary ICU admission in days, mean ± SD^a^	9.3 ± 12.2	17.2 ± 26.0	0.144
Any organ failure	29 (100 %)	25 (83.3 %)	0.067
Respiratory failure	29 (100 %)	24 (80 %)	**0.035**
Shock requiring vasopressors	27 (93.1 %)	19 (63.3 %)	**0.015**
Renal failure	24 (82.8 %)	15 (50 %)	**0.017**
Known bowel ischemia	9	2	**0.039**
Mortality	11	11	1.0
Days between ICU admission and first IAP measurement, median (IQR)	1 (0–26)	N/A	
Highest IAP in mmHg, mean ± SD	24 ± 5	N/A	
Meets criteria for IAH^b^	29 (100 %)	N/A	
Grade I	3 (10.3 %)		
Grade II	4 (13.8 %)		
Grade III	9 (31.0 %)		
Grade IV	13 (44.8 %)		
Meets criteria for ACS^c^	13 (44.8)	N/A	
Days between ICU admission and diagnosis of ACS, median (IQR)	1 (0–13)	N/A	

*SD* standard deviation, *ICU* intensive care unit, *IQR* inter-quartile range, *IAP* intra-abdominal pressure, *IAH* intra-abdominal hypertension, *ACS* abdominal compartment syndrome

^a^Hospital admission includes ICU admission in a non-tertiary center

^b^Criteria for IAH: *grade I* sustained IAP 12–15 mmHg, *grade II* sustained IAP 16–20 mmHg, *grade III* sustained IAP 21–25 mmHg, *grade IV* sustained IAP >25 mmHg

^c^Criteria for ACS: sustained IAP >20 and new or progressive organ failure

The rates of respiratory failure, shock requiring vasopressors, and renal failure were all significantly higher in the IAP group. All patients in whom IAP was measured had IAH, and 13/29 (44.8 %) met the criteria for ACS.

Patient characteristics were compared between patients who developed ACS and those who did not (Table 2). Age, gender, and APACHE II scores upon admission were comparable between the two groups. The interval between hospital admission and tertiary ICU admission was significantly shorter in the ACS group.

**Table 2:** Comparison between patients with IAP measurements who did and did not develop ACS

	No ACS (*n* = 16)	ACS (*n* = 13)	*p*
Characteristics			
Female gender	4 (25.0 %)	3 (23 %)	1.00
Age, mean ± SD	64 ± 12	55 ± 15	0.098
APACHE II score, mean ± SD	26 ± 5	28 ± 10	0.650
Interval between hospital admission and tertiary ICU admission in days, mean ± SD	13.6 ± 14.6	3.8 ± 4.1	**0.020**
Grade of IAH			
Grade I	3 (18.8 %)	0	
Grade II	4 (25.0 %)	0	
Grade III	4 (25.0 %)	5 (38.5 %)	
Grade IV	5 (31.3 %)	8 (61.5 %)	
Any organ failure	16 (100 %)	13 (100 %)	1.00
Respiratory failure	16 (100 %)	13 (100 %)	1.00
Shock requiring vasopressors	14 (87.5 %)	13 (100 %)	0.488
Renal failure	11 (68.8 %)	13 (100 %)	**0.048**
Multiple organ failure	15 (93.8 %)	13 (100 %)	1.00
Infected pancreatic necrosis	12 (75.0 %)	9 (69.2 %)	1.00
Surgical treatment			
Radiological drainage	9 (56.3 %)	8 (61.5 %)	0.774
VARD	6 (37.5 %)	8 (61.5 %)	0.198
Necrosectomy via laparotomy	6 (37.5 %)	2 (15.4 %)	0.238
Decompression laparotomy	2 (12.5 %)	10 (76.9 %)	**<0.001**
Outcome			
Bowel perforation or fistula	6 (37.5 %)	6 (46.2 %)	0.638
Gastrointestinal ischemia or necrosis	1 (6.3 %)	8 (61.5 %)	**0.003**
Length of ICU stay in days, median (IQR)	21 (4–86)	48 (1–135)	0.116
Mortality	4 (25.0 %)	7 (53.8 %)	0.143

*SD* standard deviation, *IAH* intra-abdominal hypertension, *ACS* abdominal compartment syndrome, *VARD* video-assisted retroperitoneal debridement

### Treatment of ACS

Most patients with pancreatitis developed ACS within the first week of hospitalization. Three patients with ACS underwent conservative treatment (Table 3). One of these patients died within 2 days of admission to the ICU; autopsy revealed extensive ischemia of the small and large intestines. The other two patients recovered from ACS without surgery.

**Table 3:** Time to decompression, lactate levels, the presence of ischemia, and improvement after decompression in ACS patients

Patient	Time from diagnosis ACS to decompressive surgery (h)	Lactate pre-operative (mmol/L)	Lactate post-operative (mmol/L)	Ischemia present/absent	Improvement after decompression
1	No decompression	15 (before death)	N/A	Present (autopsy)	N/A
2	No decompression	1.3	N/A		N/A
3	No decompression	1.3	N/A		N/A
4	2	3.2	3.5	Absent	Yes, respiratory and diuresis No hemodynamic improvement
5	176	0.8	0.8	Absent	No
6	4	1.4	2.7	Absent	Yes, respiratory
7	10	6.4	4.1	Present	No, persistent high IAP. Relaparotomy: ileum resection and new stoma
8	19.5	1.6	1.5	Present	Yes, respiratory and hemodynamic. IAP persistent high.
9	23.5	1.1	1.9	Present	No
10	37	6.4	11.5	Present	Yes, respiratory. Hemodynamics very unstable, relaparotomy: sigmoid necrosis, 2 gastric perforations
11	2	9.2		Present	No, treatment withdrawal and death
12	14	8.3	9.2	Present	No, persistent high IAP
13	2	2.4	2.2	Present	No, persistent high IAP. Relaparotomy: Ischemia left colon and coecum

Decompressive laparotomy was performed in 10 patients with ACS. The mean time between hospital admittance and decompressive laparotomy was 6.5 days (SD 5.5; range 1.9–15.5 days). The range in time between diagnosis of ACS and decompressive surgery was 2–176 h, with a median of 12 h. A further two patients were reported to have undergone decompressive laparotomy but did not meet the criteria for ACS according to the WSACS. The decision to perform a decompressive laparotomy in these patients was made on clinical grounds other than IAP measurements, and they are therefore not analyzed in the ACS group. The most frequently used surgical approach was full thickness transverse subcostal laparotomy (bilateral in six cases and unilateral in one case); a midline laparotomy was performed in the other three patients. The subcostal laparotomy is a preferred approach for decompression in pancreatitis patients in this center since it provides easy access to the pancreas if necessary at a later stage. Lactate measurements were increased pre-operatively in four of seven patients with ACS and ischemia in whom decompressive surgery was performed (Table 3).

The patient with liver ischemia was managed conservatively; bowel ischemia was resected in five patients.

### Effect of decompressive laparotomy

The mean IAP before surgery was 27 ± 3 mmHg, and this decreased to 18 ± 4 mmHg immediately after decompression (*p* = 0.001). There was an improvement in the respiratory and/or hemodynamic condition immediately after decompression in four patients (Table 3). However, four patients had a persistently high IAP >20 mmHg after decompressive surgery. In three patients, decompression had been performed using a bilateral subcostal incision and in one patient with a midline laparotomy. One patient had a relaparotomy one day after decompression with resection of ischemic cecum and left colon. Afterward, IAP decreased to 17 mmHg. One patient had a necrotic ileostomy for which partial ileum resection and a new ileostomy was placed. Two patients were managed conservatively at first but required multiple relaparotomies in the course of their disease. These patients both died from sepsis and multi-organ failure secondary to SAP. One other patient required a relaparotomy one day after decompression. This patient had been too hemodynamically unstable in the operating theater to continue the operation after decompression and was stabilized in the ICU first. This patient had necrosis of the sigmoid colon and two gastric perforations.

### Intra-abdominal ischemia and outcome

Although there was significantly more shock requiring vasopressors in the group where IAP was measured (*p* = 0.015, Table 1), there was no significant difference in the occurrence of shock requiring vasopressors in ACS compared to the group without ACS (Table 2). Renal failure was present significantly more in patients with ACS (100 vs. 68.8 %, *p* = 0.048). The majority of patients in both groups developed infected pancreatic necrosis at some point during admission. Enteric fistulae and/or perforations were seen at similar rates in patients with and without ACS.

Intra-abdominal ischemia occurred in 11 of 59 patients (18.6 %). This was significantly higher in patients with ACS. In nine patients, IAP measurements had taken place; eight of these patients had ACS (Table 2). The location of ischemia varied (Table 4).

**Table 4:** Gastrointestinal ischemia in eight patients with ACS

Pt nr.	Max IAP	Macroscopy	Microscopy	Outcome
1	22	Autopsy findings: Focal ischemia on serosal and mucosal side of small bowel	Mucosa of jejunum and colon almost completely disappeared	Died 2 days after admission to the ICU
7	24	Full necrosis of the sigmoid	Transmural ischemic necrosis sigmoid	Died 67 days after decompression due to bleeding complications caused by pancreatitis
8	26	Dubious signs of ischemia of terminal ileum	Segmental chronic and acute inflammation with perforation; concordant with ischemic enteritis	Died 40 days after decompression due to sepsis and MOF
9	27	Firm necrosis of the jejunum	Severe transmural ischemic enteritis	Discharge to ward 134 days after decompression
10	27	Only necrotic remains of the sigmoid colon	Transmural necrosis	Died 13 days after decompression due to sepsis and MOF
11	29	Full black necrosis of small and large intestine	Not available	Died several hours after decompression, after withdrawal of treatment
12	32	Marbled aspect of left liver lobe	Not available	Died 47 days after decompression, due to sepsis and MOF
13	32	Patchy and full ischemia of the terminal ileum; full ischemia of cecum and left colon	ischemia ileum, cecum and left colon	Discharge to ward 16 days after decompression

*Pt nr* patient number, *ACS* abdominal compartment syndrome, *ICU* intensive care unit, *IAP* intra-abdominal pressure, *MOF* multiple organ failure

One patient died after conservative management of ACS. Autopsy revealed extensive small bowel ischemia (Table 4). The mortality in patients after decompressive surgery was 6/10 (60 %). Five of these patients (83 %) had been diagnosed with intra-abdominal ischemia upon decompressive laparotomy. Only two patients with ischemia survived until discharge from the ICU. One of these patients was subsequently discharged from the hospital and recovered fully. The other patient was transferred to another hospital and died following a neurological complication 3 months later. One patient with ACS died after supportive treatment was withdrawn after decompressive laparotomy showed extensive ischemia (Table 3). The five other patients died 28–68 days after surgery; four deaths were due to sepsis and multiple organ failure, and one death was due to bleeding complications (Table 4).

One patient with intra-abdominal ischemia had no ACS. This patient developed ischemia of the cecum one day after a laparotomy for drainage of an infected pseudocyst which was complicated by diffuse bleeding. This patient died from septic complications following multiple relaparotomies. In two patients with intra-abdominal ischemia, no IAP had been measured. One patient died from MOF within 1 day of hospitalization. Autopsy showed extensive ischemia to small intestines and colon. The other patient underwent resection of jejunum ischemia after developing acute abdominal pain one week after discharge from the ICU. This patient subsequently improved but died in hospital 1 month later due to withholding of therapy in sepsis.

## Discussion

This study found that IAH and ACS are common in patients with severe acute pancreatitis, confirming earlier series [2, 3]. As a novel finding, a high incidence of intestinal ischemia was observed in patients with pancreatitis and ACS.

IAH was present in all patients in whom IAP was measured. In previous series, IAH was found in approximately 60 % of the patients studied [2, 3]. The high rate in our study may be an overestimation resulting from selective IAP measurements in patients at risk for IAH; however, it may also be a reflection of the severity of illness of patients referred to our tertiary center.

In this series, patients were admitted to a tertiary ICU after a mean interval of 13.4 days after hospitalization for the current episode of pancreatitis. In patients with ACS, this interval was a much shorter 3.8 days. Although the exact mechanisms are not completely understood, IAH in severe acute pancreatitis is usually an early phenomenon and may have an important role in the development of early organ failure [13].

Decompression laparotomy was successful in lowering the IAP in most cases. However, the mortality rate of severe acute pancreatitis with ACS was high at 54 %. This is comparable to other series [2, 3]. Mortality was even higher in the ACS group if intra-abdominal ischemia occurred (75 %).

A causal relationship between ACS and multiple organ failure in SAP is yet to be established [14]. It is likely that IAH is involved to some extent in both development of necrosis and infection of necrosis [13]. In the current study, there was no difference in infected pancreatic necrosis rates between patients who did and did not develop ACS. Mortality in SAP typically occurs in two phases: an early phase where multiple organ failure occurs with SIRS, IAH, or ACS and a later phase where mortality is due to secondary infection and necrosis [15].

The high rate of intra-abdominal ischemia observed in patients with pancreatitis and ACS has not been reported previously. Depending on its level and the overall hemodynamic condition, IAH has been associated with bowel ischaemia [16]. The small intestine may become damaged during severe acute pancreatitis, due to microcirculation disturbance associated with loss of fluids in the “third space,” hypovolemia, splanchnic vasoconstriction, and finally ischemia–reperfusion injury [17]. Intestinal damage and loss of intestinal barrier function occur early in the course of pancreatitis [18]. IAPs were not reported in this study. IAH and ACS are associated with gut barrier dysfunction as reflected by greater systemic endotoxin exposure and procalcitonin levels [19].

The association between ACS and intestinal ischemia is further described in animal studies, showing a significant decrease in perfusion of intestinal mucosa and mesenteric arterial blood flow, after IAP was increased [7, 8]. The current study suggests that IAH may be one of the mechanisms associated with intestinal damage early in the course of SAP. Further study is warranted to confirm this hypothesis.

We fully support the WSACS recommendation that protocolized monitoring of IAP should take place in high-risk patients, such as those with severe acute pancreatitis to allow early detection and treatment of IAH and ACS. The protocol in our center has been adjusted since this retrospective series to include monitoring of IAP in all high-risk patients, and we are currently enrolling patients in a prospective study to evaluate this change. Furthermore, we recommend international guidelines for treatment of pancreatitis patients be updated to include this recommendation. Three commonly used guidelines by the British Society of Gastroenterology [20], the American College of Gastroenterology [21], and the American Gastroenterological Association [22] do not yet mention the need for routine IAP measurements.

Once IAH is observed, careful monitoring of IAP and organ functions needs to take place and measures should be taken in order to prevent further organ dysfunction and irreversible damage.

Lactate monitoring seems to be of limited value in diagnosing intestinal ischemia in ACS. In this series, it was increased in only four of seven patients with ACS and intestinal ischemia. Furthermore, increased lactate levels may be a reflection of other problems than intestinal ischemia, for example, underresuscitation (Table 3). This matches conclusions from larger series: normal or decreasing lactate values by no means rule out ischemia [23, 24].

If ACS occurs, it may be managed by non-surgical interventions to lower the pressure or to prevent a further rise. These interventions may include enteral decompression with nasogastric or rectal tubes when stomach or colon are dilated, percutaneous drainage of ascites or fluid collections, prevention of overly aggressive fluid resuscitation, and neuromuscular blockade as a temporizing measure [1].

These conservative measures were not systematically employed in this group of patients, although two patients received neuromuscular blockade in an attempt to stabilize their respiratory failure in ACS. According to this institution’s protocol, patients with pancreatitis received enteral feeding via nasogastric or nasojejunal canulas. At the time, these patients were treated, the slogan in ACS was still “open the abdomen and leave it open,” compared to current opinion where conservative management, if possible, is preferred to prevent the morbidity and mortality that may result from an open abdomen.

There are no conservative measures unique to SAP patients. However, conservative measures that may especially benefit patients with SAP are prevention of overly aggressive fluid resuscitation early in the course of the disease and percutaneous drainage of ascites or other fluid collections that may complicate SAP.

There is evidence that early decompression may improve survival [13]. However, as this retrospective series confirms, mortality is still high in this group [5]. Furthermore, an open abdomen in patients with severe acute pancreatitis is associated with significant morbidity. A reasonable strategy may be to perform swift decompression in patients with multiple organ failure and ACS when conservative management fails.

It may not be feasible to conduct large multicenter randomized trials to determine optimal treatment strategies for patients with severe acute pancreatitis and ACS; however, large, prospective observational studies may be a reasonable alternative.

## Limitations of this study

This study is limited by its retrospective design and small numbers. IAP measurements were conducted in a subgroup of patients. Therefore, it is unclear whether the results can be extrapolated to the entire population of patients with severe acute pancreatitis. Furthermore, many of the patients were referred to our center after having been hospitalized elsewhere with no records of IAP measurements.

## Conclusion

This study confirms that IAH and ACS are common findings in patients with severe acute pancreatitis. IAH may worsen the already grim course of severe acute pancreatitis. High rates of intra-abdominal ischemia were found in patients with ACS. Early recognition of this potentially treatable aggravating condition may lead to earlier intervention and hopefully improve the outcome. We advise routine measurement of IAP in all patients with severe acute pancreatitis. National and international guidelines on severe acute pancreatitis need to be updated to include IAP measurements as standard of care and to alert clinicians to this potentially lethal complication that requires swift surgical intervention if conservative measures fail.

## Key messages

- Intra-abdominal hypertension and abdominal compartment syndrome are common in patients with severe acute pancreatitis
- Intra-abdominal ischemia may complicate the course of pancreatitis and ACS
- Routine measurement of IAP is advised in all patients with severe acute pancreatitis.

## Abbreviations

ACS: Abdominal compartment syndrome
ERCP: Endoscopic retrograde cholangiopancreatography
IAP: Intra-abdominal pressure
IAH: Intra-abdominal hypertension
ICU: Intensive care unit
IQR: Inter-quartile range
MOF: Multiple organ failure
SAP: Severe acute pancreatitis
SD: Standard deviation
VARD: Video-assisted retroperitoneal debridement
WSACS: World Society of the Abdominal Compartment Syndrome
